# Clinical performance and utility of a noninvasive urine-based methylation biomarker: TWIST1/Vimentin to detect urothelial carcinoma of the bladder

**DOI:** 10.1038/s41598-024-58586-7

**Published:** 2024-04-04

**Authors:** Chanchan Zhang, Xiaohong Xu, Tao Wang, Yan Lu, Zhiheng Lu, Tuantuan Wang, Zhiwen Pan

**Affiliations:** 1 Zhejiang Cancer Hospital, Hangzhou, 310022 Zhejiang China; 2 Jiangsu MicroDiag Biomedicine Co., Ltd., Suzhou, China; 3 Hangzhou Institute of Medicine (HIM), Chinese Academy of Sciences, Hangzhou, 310022 Zhejiang China

**Keywords:** Urothelial carcinoma of the bladder, Methylation, Diagnostic test, Biomarker, Epigenetic, Cancer, Cancer screening, Tumour biomarkers, Urological cancer

## Abstract

Traditional clinical modalities for diagnosing bladder urothelial carcinoma (BUC) remain limited due to their invasive nature, significant costs, discomfort associated with cystoscopy, and low sensitivity to urine cytology. Therefore, there is an urgent need to identify highly sensitive, specific, and noninvasive biomarkers for the early detection of this neoplasm. Hypermethylated TWIST1/Vimentin promoter may be a noninvasive biomarker using urine sample. We assessed the TWIST1/Vimentin promoter methylation status in urine samples using the Methylated Human TWIST1 and Vimentin Gene Detection Kit (Jiangsu MicroDiag Biomedicine Co., Ltd., China). The samples were collected from five groups: group 1 consisted of patients with BUC, group 2 contained other patients with urologic tumors, group 3 consisted of patients with benign diseases (e.g., urinary tract infections, lithiasis, and benign prostatic hyperplasia), Group 4 included UTUC (upper tract urothelial carcinoma) patients and group5 comprised healthy individuals. The study encompassed 77 BUC patients, and we evaluated the degree of methylation of the TWIST1/Vimentin gene in their urine samples. Notably, TWIST1/Vimentin positivity was significantly elevated in comparison to groups 2, 3 and 5 (all *p* < 0.001) at a rate of 77.9%, but no significant difference was observed when compared to group 4. In the relationship between TWIST1/Vimentin methylation and clinicopathological features of BC patients from our center, we found there was no significant association between TWIST1/Vimentin status and proteinuria and/or hematuria, and hypermethylation of TWIST1 / VIM genes was found in both high and low tumor grade and in both non-muscle invasive bladder cancer (stages Tis, Ta, or T1) and muscle-invasive bladder cancer (stage T2 or above). In the multivariable analysis for cancer detection, a positive TWIST1/Vimentin methylation were significantly linked to a heightened risk of BC. Moreover, TWIST1/Vimentin promoter methylation demonstrated an ability to detect BUC in urine samples with a sensitivity of 78% and a specificity of 83%. Our findings reveal that hypermethylation of the TWIST1/Vimentin promoter occurs in bladder urothelial carcinoma, and its high sensitivity and specificity suggest its potential as a screening and therapeutic biomarker for urothelial carcinoma of the bladder.

## Introduction

Bladder cancer ranks among the top 10 most prevalent cancers globally. In 2020, the World Health Organization's International Agency for Research on Cancer issued the latest statistics regarding the global cancer burden, reporting that the year witnessed approximately 0.57 million new bladder cancer diagnoses and 0.213 million fatalities. The disease predominantly affects men, with the incidence and mortality rates among this demographic being 9.5 and 3.3 per 10 million, respectively. These rates are nearly quadruple those observed among women globally^1^. Typically, bladder cancer is prevalent in individuals aged between 50 and 70 years, with the incidence rate escalating with advancing age^2^.

Bladder cancer is divided into three types based on its pathological classifications: Urothelial carcinoma, squamous cell carcinoma, and adenocarcinoma. Notably, urothelial carcinoma represents over 90% of all bladder cancer cases. Compared to other pathological subtypes, urothelial carcinoma generally exhibits a more favorable prognosis. Urothelial carcinoma of the bladder is further stratified into muscle-invasive bladder cancer (MIBC) and non-muscle-invasive bladder cancer (NMIBC)^3^. The progression of bladder cancer typically follows a series of stages, including epithelial atypical hyperplasia, carcinoma in situ, and invasive carcinoma. The survival rate varies substantially based on the stage of the disease. Patients with NMIBC have a 5-year survival rate exceeding 70%, whereas, for those with MIBC, it is between 30 and 40%. Advanced bladder cancer presents a survival rate of less than 5%^4^. Therefore, early detection of urothelial carcinoma is crucial. Current bladder cancer screening methods are predominantly cystourethroscopy and urine cytology. Although cystoscopy is highly sensitive, it is an invasive procedure. On the other hand, cytology is a noninvasive procedure with high specificity but lacks sensitivity, which is relatively low^5^. Therefore, there is a pressing need to develop noninvasive, specific, and straightforward biomarkers to expedite the diagnosis of bladder cancer.

Epigenetic alterations, such as DNA methylation, are integral to cancer development. Studies have found distinct DNA methylation patterns associated with bladder cancer in DNA samples obtained from the urine of patients diagnosed with BC(Bladder Cancer)^6^. For instance, research by Renard et al. indicated that a urine-based TWIST1 DNA methylation test for non-muscle invasive bladder cancer (NMIBC) offers high sensitivity and specificity (> 90%)^7^. Additionally, a study by El Azzouzi M et al. using the Methylation-Specific PCR (MSP) approach on 70 tumor biopsies from Moroccan bladder cancer patients, and demonstrated that hypermethylation of the hTERT, TWIST1, VIM, and NID2 genes, occurring at frequencies of 90%, 85.71%, 67.14%, and 67.14%, respectively, is a common epigenetic phenomenon in bladder cancer. TWIST1 is part of the highly conserved family of basic helix-loop-helix (bHLH) transcription factors, playing critical roles in various stages of embryonic development and significantly facilitating tumor metastasis and primary tumor growth. Vimentin (VIM), an essential protein in the intermediate filament family, is typically expressed in cells originating from mesenchyme. Some studies suggest that VIM is linked to the progression of epithelial cancer and the prognosis of patients^8^.

Various assay kits have recently been developed to detect specific gene methylation. In this study, we utilize a Methylation Assay Kit designed explicitly for the Twist1/Vimentin gene (produced by Jiangsu MicroDiag Biomedicine Co., Ltd., China), intending to assess its diagnostic precision and clinical applicability in patients with bladder cancer.

## Patients and methods

### Patients

After approval from the institutional review board (Opinion No. IRB- [2019]31), the urine specimens of patients undergoing transurethral resection of the bladder tumor (TURBT) or cystoscopy at Zhejiang Province Cancer Hospital for suspected primary urothelial carcinoma of the bladder were prospectively collected from October 2019 to January 2020. Furthermore, patients were excluded from the cancer group if final histology revealed nonurothelial cancer tissue and then included in the control group instead.

Overall, we divided patients into five groups: the cases were group 1, and for control group 2, we used urine samples from patients with other histopathological confirmed urological malignancies- prostate, kidney cancers. Urine from patients with benign diagnoses formed control group 3(e.g., urinary tract infections, lithiasis, and benign prostatic hyperplasia). Urine from patients with histopathological confirmed upper tract urothelial carcinoma comprised control group 4 (e.g., cancers of renal pelvis, ureter). Meanwhile, urinary samples collected from healthy controls were assigned to group5.

Before TURBT or cystoscopy, the first-morning urine samples (more than 10 ml) were collected for the controls and the patients, and stored at 4˚C until further processing. According to the manufacturer's instructions of Methylated Human TWIST1 and Vimentin Gene Detection Kit (Jiangsu MicroDiag Biomedicine Co., Ltd., China), the cells were subsequently harvested by centrifugation, and genomic DNA was extracted and purified, with modifications for subsequent PCR.

### Methylation analysis

Methylation analysis of TWIST1, Vimentin in urinary cell pellet DNA was performed by MethyLight, a highly sensitive, quantitative real-time PCR assay. Methylation status was analyzed as a categorical variable (methylation positive or negative) based on the kit instructions (Jiangsu MicroDiag Biomedicine Co., Ltd., China).

The sequences of primers and probes for the two genes are listed in Table 1. The ACTB (actin beta) gene served as a reference gene. PCR cycling conditions were set up with the following program: 95 °C for 5 min, followed by 45 cycles of 95 °C for 20 s, and 63 °C for 40 s with fluorescence signal collection.

**Table 1:** Sequences of primers and probes.

Primers and probes	Sequences (5′ → 3′)
TWISTI-F	CGGTAAGAAGTTTGCGGGTTG
TWISTI—R	AAATACGCTAACGCTCCC
TWISTI—P	FAM—AGCGGCGGCGGGAGTT—MGB
Vimentin—F	TAATCGGCGGGATAGTAGGG
Vimentin—R	GCGCCTCTATCCATCGACTT
Vimentin—P	FAM—CGTCGTTTCGTAATTTTCG—MGB
ACTB-F	GTGATGGAGGAGGTTTAGTAAGTT
ACTB-R	CCAATAAAACCTACTCCTCCCTTAA
ACTB-P	VIC-ACCACCACCCAACACACAA-MGB

The criteria for the interpretation of positive methylation results were as follows: A Cp-value ≤ 40.49 was deemed positive for Twist1 methylation; A Cp-value ≤ 41.63 was deemed positive for Vimentin methylation; A positive test result was defined as the positive detection of at least one of the two genes being assayed.

### Statistical analyses

Descriptive statistics, Pearson's chi-square test, and multivariable logistic regression analysis were used to analyze data using SPSS 25.0. T-tests and analysis of variance were used to compare general characteristics between patients and controls.

### Ethics approval and consent to participate

The Zhejiang Cancer Hospital Ethics Committee granted ethical approval (Opinion No. IRB- [2019]31), all research was performed in accordance with relevant guidelines/regulations, and informed consent was obtained from all participants and/or their legal guardians.

## Results

Amongst the 77 patients in group1, 65 were male and 12 were female with a sex ratio of 5. The mean age of patients was 65 years, ranging from 57 to 71.5 years. Based on the WHO histological grading system classification criteria for urothelial cancer in 2004, we categorized 35 specimens as a low-grade urothelial carcinoma (G1), and 35 as high-grade urothelial carcinoma (G2), with seven specimens of unknown grade. Furthermore, according to the TNM staging method proposed by the Union for International Cancer Control (UICC), we categorized 30 patients as having non-muscle invasive bladder cancer (stages Tis, Ta, or T1),11 patients as having muscle-invasive bladder cancer (stage T2 or above), and 36 patients with an Tx stage. Group 2(People with other urological malignancies) consists of 34 kidney malignancies and 47 prostatic origin malignancies, possess 71 male and 10 female. The benign urological diseases (control group3) included 11 cases of prostatic diseases, 1 cases of kidney disease, 1 renal pelvis disease and 6 bladder diseases. Table 2 shows more detailed in demographics (sex, age) and clinicopathological characteristics (e.g., tumor stage, Invasion depths) of group 1 to group 5.

**Table 2:** Distribution of the clinicopathological characteristics of the patients and controls included in the study.

	1. Patients	2. People with other urological malignancies	3. People with benign disease	4. UTUC	5. Healthy control
Total	77	81	19	26	28
Gender					
Male	65 (84.4%)	71 (87.7%)	17 (89.5%)	19(73.1%)	10(35.7%)
Female	12 (15.6%)	10 (12.3%)	2 (10.1%)	7(26.9%)	18(64.3%)
*P* value		0.557	0.844	0.162	< 0.001
Age					
≥ 60	56(72.7%)	55(67.9%)	15(78.9%)	21(80.8%)	0
< 60	21(27.3%)	26(32.1%)	4(21.1%)	5(19.2%)	28
Mean	65.12	64.82	65.26	66.86	34.71
Median	65	67	64	69	32.5
M (P_25_,P_75_)	(57,71.5)	(58,71)	(61,70)	(63,74)	(25,42.25)
*P* value		0.981	0.599	0.814	< 0.001
Lesion site					
Prostate		47(58%)	11(58%)		
Kidney		34(42%)	1(5%)		
Renal pelvis		0	1(5%)		
Bladder		0	6(32%)		
Tumor stage					
0, I	30	24		5	
II	4	10		6	
III	6	9		1	
IV	1	11		4	
Unknown	36	27		10	
*P* value		0.001		0.005	
Invasion depths					
Non-muscle invasive	30				
Muscle-Invasive	11				
Tx	36				
Grade					
G1	35				
G2	35				
Unknown	7				

We also evaluated the relationship between TWIST1/Vimentin methylation and clinicopathological features of BC patients from our center (Table 3). Amongst the 77 patients, 64 were naive patient, 13 were tumor recurrence. Hematuria was present in 46 cases (60%), 29 (38%) cases had proteinuria. And the methylation positivity of them was not significantly different compared with the Hematuria, proteinuria negative control, respectively. Meanwhile, we observed a noticeable correlation between the methylation status of the TWIST1/Vimentin promoter and physiological age. The TWIST1/Vimentin positivity rate was higher in patients older than 60 years compared to those under 60. Hypermethylation of TWIST1 / VIM genes was found in both high and low tumor grade and in both non-muscle invasive bladder cancer (stages Tis, Ta, or T1) with 38.3% and muscle-invasive bladder cancer with 16.7% (stage T2 or above), however, with no significant difference. Besides, there are also no significant difference between the methylation status of the TWIST1/Vimentin promoter and sex.

**Table 3:** The relationship between the clinicopathologic characteristics of patients with and Methylation analysis of Twist1/Vimentin.

Clinical characteristic	Total	Methylation analysis of Twist1/Vimentin		Χ^2^	*P*
	N	Negative	Positive		
	77	17	60		
Age					0.001^a^
≥ 60	56	7	49	9.003	0.003^b^
< 60	21	10	11		
Gender				0.000	1.000
Male	65	14	51		
Female	12	3	9		
Tumor recurrence				0.074	0.786^b^
Yes	13	2	11		
No	64	15	49		
Hematuria				0.825	0.364
Yes	46	9	37		
No	24	7	17		
Unknown	7	1	6		
Albuminuria				0.096	0.757
Yes	29	6	23		
No	42	10	32		
Unknown	6	1	5		
Tumor stage				2.879	0.371
0, I	30	7	23		
II	4	0	4		
III, IV	7	0	7		
Unknown	36	10	26		
Invasion depths				1.285	0.526
Non-muscle invasive	30	7	23		
Muscle-invasive	11	1	10		
Tx	36	9	27		
Tumor Grade				3.216	0.196
G1	35	9	26		
G2	35	5	30		
Unknown	7	3	4		

^a^Independent sample *t* test; ^b^adjusted Chi-Square test.

The results of a reduced penalized multivariable logistic regression model for BUC are presented in Table 4. After adjusting for confounding factors as age, sex, positive TWIST1/Vimentin methylation was significantly associated with an increased risk of BUC (OR = 11.621, 95% CI 4.012–33.621, p < 0.001).

**Table 4:** Logistic regression model of risk factors for BUC.

	Patients	People with benign diseases and healthy controls	OR (95% CI)	*P* value
Gender				
Male	65	27	1	
Female	12	20	0.287 (0.086–0.961)	0.043
Age, median, IQR	65, (57,71.5)	32.5, (25,42.25)	1.056 (1.018–1.094)	< 0.01
Twist1/Vimentin				
Negative	17	39	1	
Positive	60	8	11.621 (4.012–33.621)	< 0.001
AUC (95% CI)		0.805 (0.722–0.887)		< 0.001

**Figure 1:**
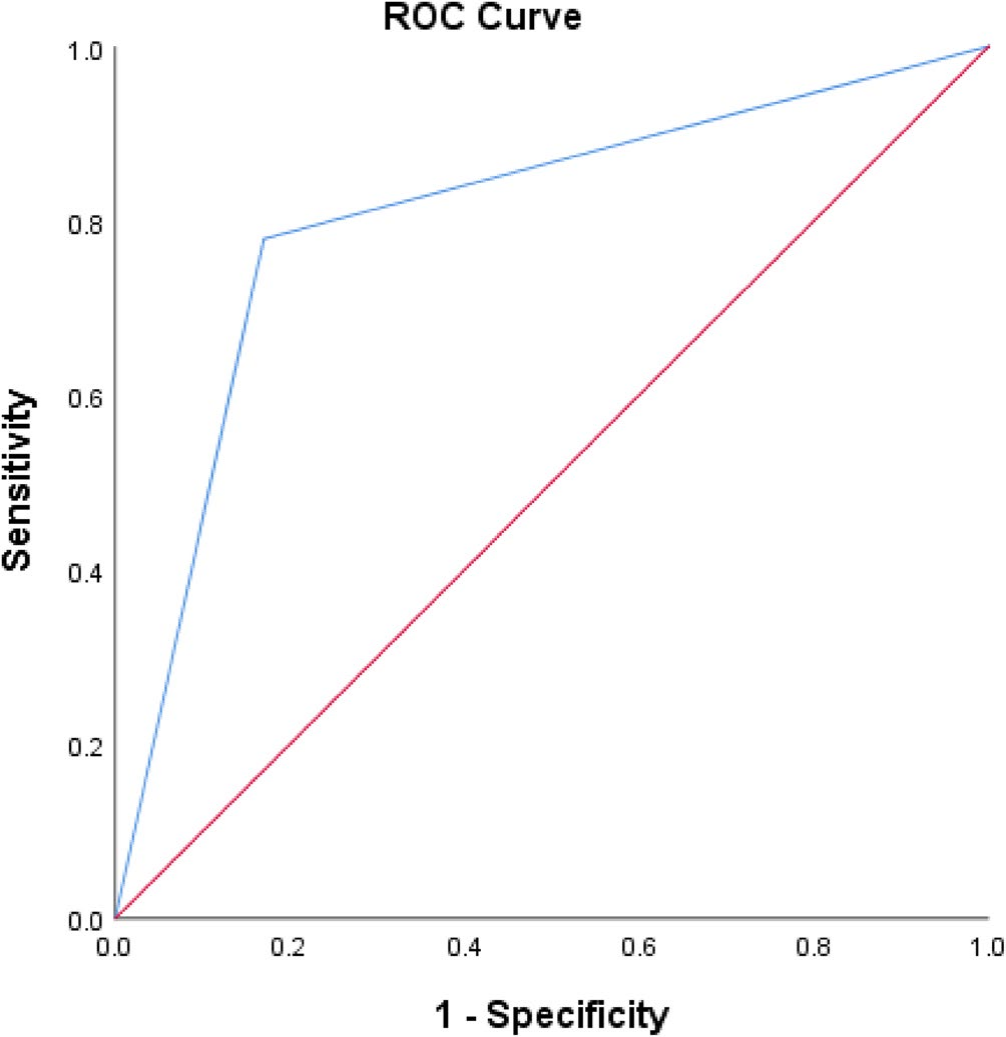
ROC curves analysis of Twist1/Vimentin methylation status between BUC patients and healthy/Benign individuals.

Additionally, we drew the ROC curve of predicted probability and calculated the AUC value (Fig. 1). Based on our study, TWIST1 and Vimentin promoter methylation could distinguish bladder cancer patients from healthy/benign subjects with 78% sensitivity and 83% specificity in urine samples.

Table 5 contains results when defining the assay as positive when the methylated count for either gene exceeded the threshold ("believe the positive"). The positive rate of cohorts 1 to 5 was respectively 77.9%, 18.5%, 15.8%, 92.3%, 17.9%. Group 1 statistically different from groups 2, 3 and 5 (*p* < 0.001), but no differences were observed in the methylation of TWIST1/Vimentin between group 1 and group 4.

**Table 5:** The results of methylation among patients and other controls.

	Total	Methylation analysis of TWIST1		Χ^2^	*P*
		Negative (%)	Positive (%)		
1. Patients	77	17 (22.1)	(77.9)		
2. People with other urological malignancies	81	66 (81.5)	15 (18.5)	55.865	< 0.001
3. People with benign diseases	19	16 (84.2)	3 (15.8)	26.08	< 0.001
4. UTUC	26	2 (7.7)	24 (92.3)	2.455	0.117
5. Healthy control	28	23 (82.1)	5 (17.9)	31.413	< 0.001

## Discussion

Optimal clinical biomarkers for tumors ideally should be noninvasive (like blood-based or urine-based biomarkers) and demonstrate satisfactory specificity and sensitivity. However, none of the biomarkers or tests individually meet these criteria. Specific markers, such as NMP-22 and BTA, have received approval from the US Food and Drug Administration (FDA) to diagnose and monitor bladder cancer. These markers display a higher sensitivity, particularly for low-grade tumors, but their specificity is lower than that of urine cytology^9^.

Renard et al.^7^ introduced a new avenue for exploring biomarkers for bladder urothelial carcinoma (BUC). They reported that the TWIST1 promoter exhibited excellent sensitivity and specificity in detecting BUC in urine samples, suggesting that aberrant DNA methylation could serve as a potential molecular tumor biomarker for BUC detection. In mammalian genomes, DNA methylation primarily occurs at CpG dinucleotides. Interestingly, in the promoter region, CpG islands—areas of high CpG dinucleotide density—often lack DNA methylation^10^. Consequently, abnormal methylation in the gene promoter region can lead to the inactivation of tumor suppressor genes, thereby acting as a driving force for neoplastic transformation.

In this study, we evaluated the methylation status of the promoter regions of TWIST1, VIM in urine samples from patients with BC. And the results show us that the panel likely to provide great clinical benefit. Our study demonstrates that the sensitivity and specificity of TWIST1/Vimentin promoter methylation in urine samples for differentiating bladder urothelial carcinoma (BUC) from benign diseases and healthy controls reached 78% and 83%, respectively.

In this study, among the 77 bladder cancer patients, the TWIST1/Vimentin positivity was significantly high, reaching 77.9%, whereas, in individuals with other urological malignancies, the positivity rate was only 18.5%. For benign and healthy controls, it stood at 15.8% and 17.9%. Our findings underscore that TWIST1/Vimentin promoter hypermethylation is significantly associated with the risk of bladder cancer, corroborating several prior studies^11,12^.

Moreover, in our study, there was no significant association between TWIST1/Vimentin status and proteinuria and/or hematuria (Table 3). We therefore assume that this kit is not affected by hematuria and proteinuria. In addition, hyper-methylation of this gene panel was recorded in all BUC grades and stages, which may make up for the lower sensitivity of urine exfoliative cytological examination.

Interestingly, our study observed a noticeable correlation between the methylation status of the TWIST1/Vimentin promoter and physiological age. The TWIST1/Vimentin positivity rate was higher in patients older than 60 years compared to those under 60. It is widely recognized that age significantly impacts DNA methylation. Numerous studies have shown that aging is associated with changes in tumor suppressor genes and overall genome hypomethylation^13,14^. However, the precise mechanisms through which age induces hypermethylation of the TWIST1/Vimentin promoter remain unclear. It could be linked to increased expressions of DNMTs and enhanced DNA methylation in these regions or a rise in detected tumor cells in urine samples with aging^15^.

Methylation of CGIs in the TWIST1 promoter region has been identified in various cancers, such as gastric, breast, colorectal, and lung cancers^16–18^. The down-regulation of Vimentin gene function in pre-malignant lesions (e.g., adenoma) can be attributed to an epigenetic modification through the methylation of the Vimentin promoter^19^. However, reports on TWIST1/Vimentin methylation in urothelial carcinomas of the upper urinary tract or prostate cancer are almost non-existent. Our data indicate that the TWIST1/Vimentin positivity in urothelial carcinoma cases exceeds that in other urological malignancies (prostate cancer, kidney cancer, urachal carcinoma), with the difference being Interesting, no significant methylation differences were found between urothelial carcinoma cases and upper tract urothelial carcinoma (UTUC) cases, despite their distinct etiologies and pathogenesis, as both are derived from urothelial tumors^20^.

## Conclusion

Overall, our study suggests that the methylation status of the TWIST1/Vimentin promoter, functioning as urinary methylation markers, provides a valuable noninvasive strategy for the sensitive and specific identification of bladder urothelial carcinoma (BUC), achieving a sensitivity of 73% and a specificity of 83%. Additionally, we utilized the Methylated Human TWIST1 and Vimentin Gene Detection Kit (produced by Jiangsu MicroDiag Biomedicine Co., Ltd., China) in our research. This kit establishes the combined detection of methylation in these two genes as a biomarker and facilitates early detection of bladder cancer using urine samples. It offers the benefits of high sensitivity, high specificity, and non-invasiveness. And it seems unaffected by hematuria and proteinuria, allowing for primary screening for BC in patients with hematuria or proteinuria. However, this study has limitations: The sample size was relatively small. Our findings require further validation through more extensive cohort studies and follow-up studies before broadly integrating them into clinical applications.

## Data Availability

All datasets involved in this study are included in the article or Additional files, and further inquiries can be directed to the corresponding author.
